# Preoperative inflammation-based immune prognostic nomogram in bladder cancer: a multicenter study

**DOI:** 10.3389/fonc.2025.1644747

**Published:** 2026-01-14

**Authors:** Runlin Feng, Jian Zhang, Yanping Tao, Jian Hou, Wenlin Tai

**Affiliations:** 1 Department of Pathology, The Second Affiliated Hospital, Kunming Medical University, Kunming, Yunnan, China; 2 Yunnan Key Laboratory of Stomatology & Department of Oral and Maxillofacial Surgery, The Affiliated Stomatology Hospital, Kunming Medical University, Kunming, Yunnan, China; 3 Department of Emergency Medicine, Kunming Third People’s Hospital, Kunming, Yunnan, China; 4 Department of Urology, The First Affiliated Hospital, Kunming Medical University, Kunming, Yunnan, China; 5 Department of Laboratory Medicine, The Second Affiliated Hospital, Kunming Medical University, Kunming, Yunnan, China

**Keywords:** bladder cancer, radical cystectomy, systemic inflammation, NLR, prognostic nomogram, overall survival, immune microenvironment, tumor inflammation

## Abstract

**Background:**

The systemic inflammatory response has been increasingly recognized as a crucial determinant of tumor progression and prognosis across various malignancies, including bladder cancer (BCa). Preoperative inflammation-based indices, which reflect the dynamic interaction between the tumor and host immune system, offer promising prognostic insights. However, existing studies have largely overlooked the synergistic integration of these indices with histopathological factors into a validated clinical tool for individualized survival prediction following radical cystectomy (RC).

**Methods:**

We conducted a retrospective multicenter study involving 1,387 BCa patients who underwent RC at two tertiary hospitals in Yunnan, China, from 2014 to 2024. Key preoperative systemic inflammatory indices—including neutrophil-to-lymphocyte ratio (NLR), platelet-to-lymphocyte ratio (PLR), systemic immune-inflammation index (SII), and systemic inflammation response index (SIRI)—were extracted alongside clinical and pathological variables such as AJCC stage, perineural invasion (PNI), and lymphovascular invasion (LVI). Variable selection was performed via LASSO regression, and independent predictors were identified using multivariate Cox regression. A prognostic nomogram was developed and validated through concordance index (C-index), time-dependent ROC curves, calibration plots, and decision curve analysis (DCA).

**Results:**

The final nomogram included five independent predictors: NLR, PLR, AJCC stage, PNI, and LVI. The model demonstrated robust discrimination with C-indices of 0.78 in the training cohort and 0.72 in the external validation cohort. AUC values consistently exceeded 0.75 at 1-, 3-, and 5-year timepoints. Calibration plots showed excellent agreement between predicted and observed outcomes, and DCA confirmed meaningful clinical benefit across various threshold probabilities.

**Conclusion:**

This study presents a validated, inflammation-based prognostic nomogram that combines preoperative hematologic indices with pathological features to predict overall survival in BCa patients undergoing RC. The model is non-invasive, cost-effective, and easy to implement using routine clinical data. Its strong predictive performance supports its application in preoperative counseling, individualized surveillance strategies, and decision-making regarding adjuvant therapy, contributing to more precise and personalized management in bladder cancer care.

## Introduction

Bladder cancer (BCa) ranks among the most prevalent malignancies of the urinary system, with its global incidence placing it tenth among all cancers. The burden of disease continues to rise, particularly in developing countries and elderly male populations. According to the 2024 Global Burden of Disease (GBD) study, over 570,000 new cases and more than 200,000 deaths from BCa occur globally each year, highlighting its substantial health impact (1, 2). Radical cystectomy (RC) remains the standard treatment for patients with muscle-invasive bladder cancer (MIBC) and certain high-risk non-muscle-invasive bladder cancer (NMIBC), offering substantial survival benefits (3). However, despite advancements in surgical techniques, the five-year overall survival (OS) rate ranges between 30% and 60%, revealing significant variability between patients (4). This suggests that traditional staging systems, such as TNM classification and histological grading, may not fully capture the biological heterogeneity influencing individual prognosis.

Systemic inflammation has emerged as a critical driver of tumorigenesis, contributing to tumor proliferation, metastasis, and immune evasion mechanisms (5, 6). In the tumor microenvironment of BCa, inflammatory mediators promote leukocyte recruitment, neovascularization, and immunosuppressive signaling, which foster tumor growth and progression (7, 8). Inflammation-based markers derived from blood, including the neutrophil-to-lymphocyte ratio (NLR), platelet-to-lymphocyte ratio (PLR), systemic immune-inflammation index (SII), and systemic inflammation response index (SIRI), have shown prognostic value across various cancers, such as laryngeal, gastrointestinal, thyroid, breast, and renal malignancies (9–14).

In BCa, elevated preoperative levels of NLR and PLR are associated with increased recurrence, progression, and reduced immunotherapy efficacy. A meta-analysis demonstrated that an NLR > 3.0 significantly correlates with poor postoperative OS in RC patients (15). Furthermore, SII and SIRI have shown promising predictive capabilities in high-risk NMIBC populations treated with BCG (16). Elevated PLR is thought to reflect platelet-mediated secretion of tumor-promoting cytokines, potentially facilitating immune evasion and metastatic spread (17). Alongside systemic markers, pathological features such as perineural invasion (PNI) and lymphovascular invasion (LVI) have emerged as critical indicators of micrometastatic potential. However, their potential interaction with inflammation and their joint prognostic impact remain underexplored (18, 19). Despite their potential, inflammation-based indices are often constrained by small sample sizes, poor model design, and insufficient external validation. Few studies have integrated multiple inflammatory scores with classical pathological parameters into a clinically applicable tool for individualized prognosis.

To address these gaps, we conducted a multicenter retrospective study involving real-world data from two tertiary hospitals in Yunnan Province. We evaluated the prognostic significance of preoperative NLR, PLR, SII, and SIRI in patients undergoing RC. These indices were integrated with AJCC stage, PNI, and LVI to develop a quantitative nomogram. The model’s predictive performance and clinical utility were assessed through the concordance index (C-index), time-dependent ROC curves, calibration plots, and decision curve analysis (DCA). The primary outcome of our study was the development and validation of an inflammation-based immune prognostic nomogram that predicts OS in bladder cancer patients undergoing RC. The nomogram integrates preoperative systemic inflammatory indices, including NLR, PLR, SII, and SIRI, with key pathological factors such as AJCC stage, PNI, and LVI. Secondary outcomes included the identification and evaluation of independent prognostic factors for OS, including NLR, PLR, AJCC stage, PNI, and LVI, which were confirmed as significant predictors in multivariate Cox regression analysis. Additionally, the nomogram’s predictive ability was validated using various statistical metrics, including time-dependent ROC curves, calibration plots, and DCA, demonstrating its clinical applicability and strong performance across multiple datasets. Our goal was to establish a practical tool for individualized risk stratification and facilitate precision-guided management in bladder cancer care.

## Materials and methods

### Patient selection

**Figure 1:**
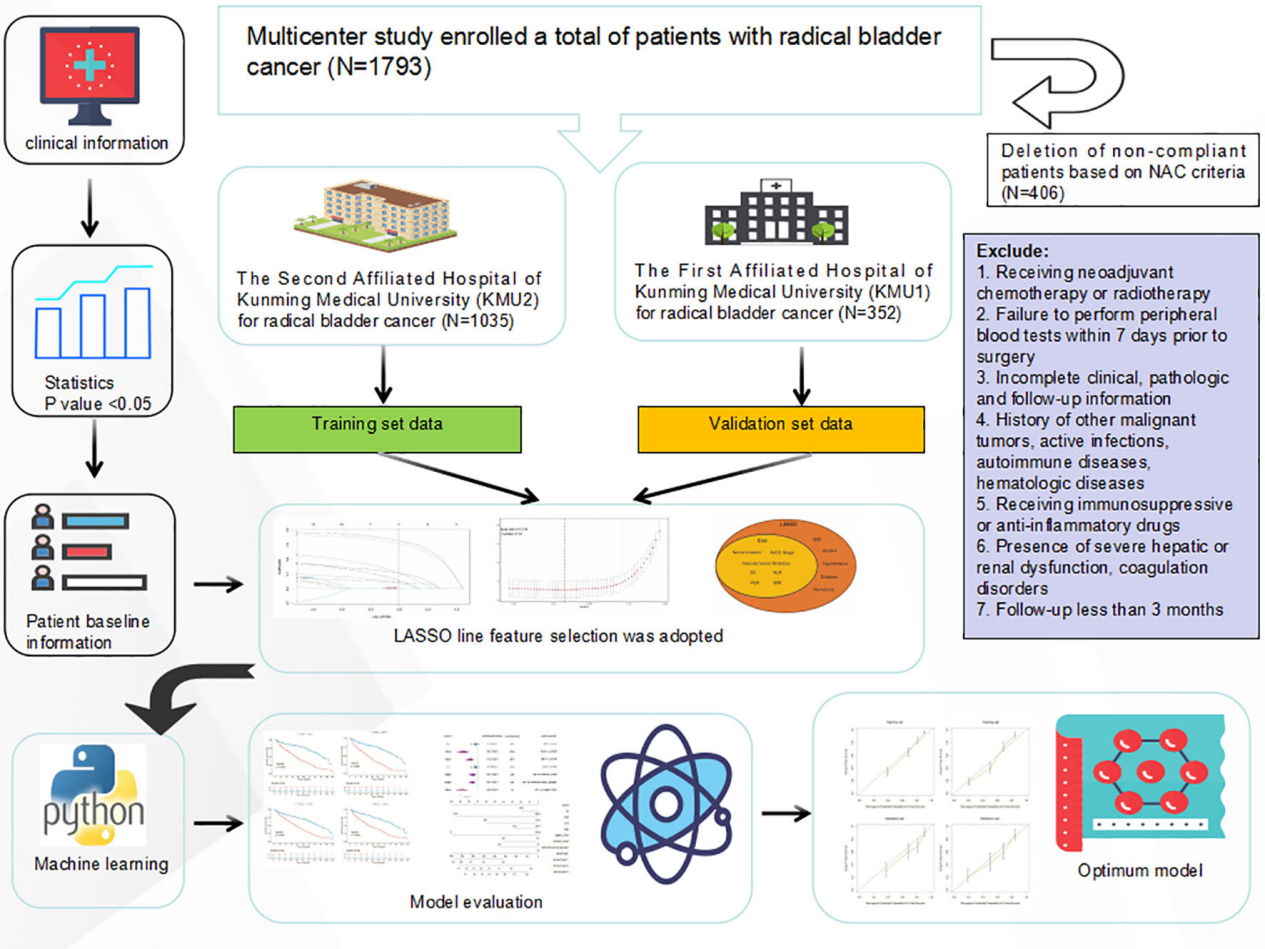
The workflow of this study.

This retrospective multicenter study involved 1,387 patients with histologically confirmed bladder urothelial carcinoma who underwent radical cystectomy from January 2014 to December 2024.A total of 1,035 patients received treatment at the Second Affiliated Hospital of Kunming Medical University, while 352 patients were treated at the First Affiliated Hospital. Eligible patients were required to have undergone primary radical cystectomy without receiving neoadjuvant chemotherapy or radiotherapy, and to have preoperative peripheral blood tests available within seven days prior to surgery. Participants were included only if they had comprehensive clinical, pathological, and follow-up data. Patients were excluded if they had a history of other malignancies, active infections, autoimmune diseases, hematologic disorders, or were receiving immunosuppressive or anti-inflammatory medications at the time of surgery. Additional exclusion criteria included severe hepatic or renal dysfunction, coagulation disorders, or incomplete follow-up of less than three months. The Ethics Committee of Kunming Medical University granted approval for this study (Approval No.).Shen-PJ-Ke-2024-199) waived the informed consent requirement due to the study’s retrospective nature. A study flow chart is shown in **Figure 1**.

### Definition of systemic inflammatory indices

Systemic inflammatory indices were calculated based on preoperative peripheral blood samples collected within 7 days prior to surgery. This study evaluated four inflammation-related biomarkers: the systemic immune-inflammation index (SII), calculated as platelet count multiplied by neutrophil count divided by lymphocyte count; the neutrophil-to-lymphocyte ratio (NLR), as the ratio of neutrophil count to lymphocyte count; the platelet-to-lymphocyte ratio (PLR), as platelet count divided by lymphocyte count; and the systemic inflammation response index (SIRI), as neutrophil count multiplied by monocyte count divided by lymphocyte count. All hematologic parameters were obtained using an automated hematology analyzer (Mindray BC-6900), with values expressed in units of 10^9^/L. The calculations and data entry were independently performed by two trained investigators to ensure accuracy and consistency across the dataset.

### Data collection and quality control

Two independent researchers extracted clinical and pathological data from hospital information systems. This included demographic details (age, sex, BMI, smoking and drinking history, comorbidities), tumor characteristics (TNM stage, grade, lymphovascular invasion, perineural invasion), and survival outcomes. Tumor staging followed the 8th edition of the AJCC TNM system. Overall survival (OS) refers to the duration from surgery to death from any cause or the most recent follow-up. Data abstraction and entry were independently performed by two blinded investigators using a standardized protocol. Any discrepancies were resolved by consensus. For variables with missing values of less than 5%, multiple imputation was employed to reduce bias. To ensure data integrity, patients with significant missing information were excluded from the analysis.

### Data splitting for model validation

In this study, the data were separated into training and validation cohorts to enhance the external validation of the model. This approach allows for the assessment of the model’s generalizability, ensuring that it can accurately predict outcomes across different patient populations beyond the training cohort. By using an independent validation set, we can evaluate the model’s robustness and its ability to apply to new, unseen data, thus minimizing the risk of overfitting. This separation is a standard practice in nomogram development, particularly in multicenter studies. The validation cohort serves as a test to ensure that the model’s predictive performance remains consistent across various datasets. In our study, this methodology ensures that the model is not overly tailored to the characteristics of the training cohort and can be generalized to other clinical settings. Through external validation, we demonstrate that the nomogram provides reliable and applicable predictions for bladder cancer patients undergoing radical cystectomy, even when applied to diverse and independent cohorts.

### SII and SIRI inclusion

Despite the lack of statistical significance for SII and SIRI in the multivariate analysis of this study, they were still included in the nomogram. The main reason for their inclusion is their widespread use in existing literature and their potential clinical relevance. Although these markers did not show independent predictive value in this study, combining inflammation-related biomarkers provides a more comprehensive perspective for the prognosis of bladder cancer patients. To enhance the inclusivity of the model and further validate their clinical applicability in bladder cancer, SII and SIRI were incorporated. This decision also provides a foundation for future research, aiming to improve predictive accuracy and clinical guidance by integrating more inflammation-related biomarkers.

### Python in machine learning

In this study, we used Python’s scikit-learn library for machine learning, particularly to implement LASSO regression for feature selection. LASSO regression is a widely used feature selection method, especially for high-dimensional datasets, allowing the identification of the most relevant prognostic variables from a wide range of clinical and pathological factors. We used the LassoCV function for cross-validation to select the optimal regularization parameter (λ). Then, using the selected λ value, we identified variables most significantly associated with the prognosis of bladder cancer patients following radical cystectomy, including NLR, PLR, AJCC stage, PNI, and LVI. These variables were ultimately incorporated into the nomogram. Python played a key role in the variable selection and model development process, ensuring the efficiency and accuracy of the data analysis.

### Statistical analysis

Statistical analyses were performed using R (v4.2.2), SPSS (v27.0.1), and GraphPad Prism (v10.3.1).Receiver operating characteristic (ROC) curve analysis, utilizing the Youden index, was employed to establish optimal cut-off values for SII, NLR, PLR, and SIRI. Patients were categorized into early-stage (≤T2N0M0) and advanced-stage (>T2 and/or N+/M+) groups based on AJCC staging. Categorical variables were assessed with chi-square or Fisher’s exact tests, while continuous variables were evaluated using the Mann–Whitney U test. Kaplan–Meier survival analysis with the log-rank test evaluated OS differences, while LASSO regression identified prognostic variables. Variables identified by LASSO were further evaluated in univariate and multivariate Cox proportional hazards models. A nomogram was developed using independent predictors to estimate 1-, 3-, and 5-year overall survival (OS). The model’s discrimination ability was assessed through the concordance index (C-index) and time-dependent ROC curves. Calibration curves assessed the agreement between predicted and observed survival probabilities. Tenfold cross-validation was used for internal validation, and an independent cohort was used for external validation. Clinical utility was evaluated using decision curve analysis (DCA).

## Results

### Patient cohort characteristics and inflammatory marker stratification

The study included 1,387 patients with histologically confirmed bladder cancer who underwent radical cystectomy, with 1,035 in the training cohort and 352 in the external validation cohort. **Table 1** presents a comparison of baseline demographic and clinicopathological characteristics between the two cohorts. No statistically significant differences were found in age distribution, sex, or AJCC stage (all p > 0.05), indicating comparability and suitability for subsequent model development and validation.

**Table 1:** Clinical characteristics and tumor staging statistics of patients with radical bladder cancer in training and validation cohorts.

Variables	Level	Overall(n=1387)	Training(n=1035)	Validation(n=352)	*P*-value
Gender (%)	Female	215 (15.5)	153 (14.8)	62 (17.6)	0.237
	Male	1172 (84.5)	882 (85.2)	290 (82.4)	
Age (%)	<=60	476 (34.3)	359 (34.7)	117 (33.2)	0.668
	>60	911 (65.7)	676 (65.3)	235 (66.8)	
Smoke (%)	No	714 (51.5)	528 (51.0)	186 (52.8)	0.596
	Yes	673 (48.5)	507 (49.0)	166 (47.2)	
Alcohol (%)	No	972 (70.1)	720 (69.6)	252 (71.6)	0.516
	Yes	415 (29.9)	315 (30.4)	100 (28.4)	
BMI (%)	<=25	1102 (79.5)	814 (78.6)	288 (81.8)	0.232
	>25	285 (20.5)	221 (21.4)	64 (18.2)	
Diabetes (%)	No	1237 (89.2)	922 (89.1)	315 (89.5)	0.91
	Yes	150 (10.8)	113 (10.9)	37 (10.5)	
Hypertensive (%)	No	998 (72.0)	741 (71.6)	257 (73.0)	0.658
	Yes	389 (28.0)	294 (28.4)	95 (27.0)	
Hematuria (%)	No	223 (16.1)	167 (16.1)	56 (15.9)	0.987
	Yes	1164 (83.9)	868 (83.9)	296 (84.1)	
Renal cyst (%)	No	775 (55.9)	574 (55.5)	201 (57.1)	0.635
	Yes	612 (44.1)	461 (44.5)	151 (42.9)	
Radical prostatectomy (%)	No	626 (45.1)	465 (44.9)	161 (45.7)	0.84
	Yes	761 (54.9)	570 (55.1)	191 (54.3)	
Recurrence (%)	No	1325 (95.5)	990 (95.7)	335 (95.2)	0.819
	Yes	62 ( 4.5)	45 ( 4.3)	17 ( 4.8)	
Nerve invasion (%)	No	996 (71.8)	740 (71.5)	256 (72.7)	0.708
	Yes	391 (28.2)	295 (28.5)	96 (27.3)	
Vascular tumor thrombus (%)	No	838 (60.4)	621 (60.0)	217 (61.6)	0.629
	Yes	549 (39.6)	414 (40.0)	135 (38.4)	
AJCC Stage (%)	<=II	834 (60.1)	626 (60.5)	208 (59.1)	0.691
	>II	553 (39.9)	409 (39.5)	144 (40.9)	
SII (%)	<=1005	662 (47.7)	502 (48.5)	160 (45.5)	0.354
	>1005	725 (52.3)	533 (51.5)	192 (54.5)	
NLR (%)	<=6.22	850 (61.3)	640 (61.8)	210 (59.7)	0.509
	>6.22	537 (38.7)	395 (38.2)	142 (40.3)	
PLR (%)	<=198	761 (54.9)	569 (55.0)	192 (54.5)	0.938
	>198	626 (45.1)	466 (45.0)	160 (45.5)	
SIRI (%)	<=2.53	790 (57.0)	595 (57.5)	195 (55.4)	0.534
	>2.53	597 (43.0)	440 (42.5)	157 (44.6)	

BMI (Body Mass Index): Weight/Height^2^; SII (Systemic Immune-Inflammation Index): Platelets*Neutrophils/Lymphocytes; NLR (Neutrophil-to-Lymphocyte Ratio): Neutrophils/Lymphocytes; PLR (Platelet-to-Lymphocyte Ratio): Platelets/Lymphocytes; SIRI(Systemic Inflammatory Response Index): Neutrophils*Monocytes/Lymphocytes.

In the training cohort, 882 patients (85.2%) were male, and 153 (14.8%) were female. Patients aged over 60 years accounted for 65.3% (676/1035), and 60.5% (626/1035) were diagnosed with AJCC stage ≤II disease. In the validation cohort, 290 patients (82.4%) were male, and 62 (17.6%) were female; 66.8% (235/352) were over 60 years of age, and 59.1% (208/352) had stage ≤II tumors. ROC analysis was performed to identify optimal cut-off values for systemic inflammatory indices using the Youden index. The resulting thresholds were 1005 for SII, 6.22 for NLR, 198 for PLR, and 2.53 for SIRI. Based on these thresholds, 533 patients (51.5%) in the training cohort had SII >1005, 395 (38.2%) had NLR >6.22, 466 (45.0%) had PLR >198, and 440 (42.5%) had SIRI >2.53.In the validation cohort, 192 patients (54.5%) had SII >1005, 142 (40.3%) had NLR >6.22, 160 (45.5%) had PLR >198, and 157 (44.6%) had SIRI >2.53.The median follow-up duration was 31.0 months (IQR 12.8–62.1) for the training cohort and 28.0 months (IQR 13.0–54.3) for the validation cohort. The similar distributions of inflammatory indices and clinicopathological features across both cohorts ensure the reliability of the modeling and validation processes.

### Identification of prognostic factors via LASSO regression and univariate Cox analysis

**Figure 2:**
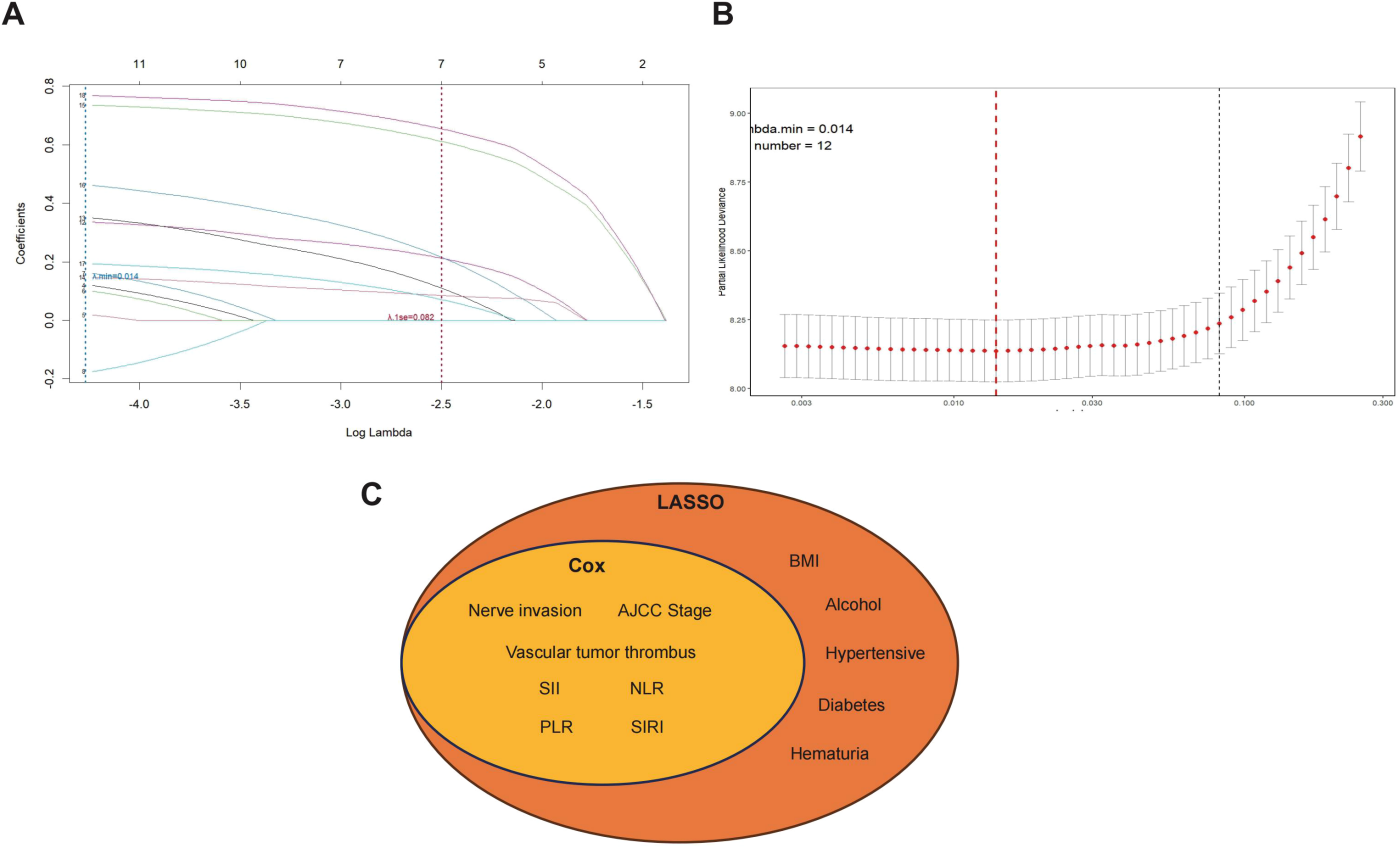
Selection of prognostic features using LASSO regression and univariate Cox analysis. **(A)** LASSO coefficient profiles of 18 candidate variables plotted against the log(λo sequence. Each curve represents the trajectory of a coefficient as a function of the regularization parameter. **(B)** 10-fold cross-validation for tuning parameter selection in the LASSO model. The optimal λ value was determined using the 1-standard-error rule, yielding 12 variables with non-zero coefficients. **(C)** Venn diagram illustrating the overlap between prognostic features identified via LASSO regression and univariate Cox analysis. Seven variables, including nerve invasion, vascular tumor thrombus, AJCC stage, SII, NLR, PLR, and SIRI, were retained in both models and selected as key predictors for multivariate analysis.

We employed least absolute shrinkage and selection operator (LASSO) regression on 18 clinical and pathological variables to determine independent predictors of overall survival (OS) in bladder cancer patients undergoing radical cystectomy. The optimal penalty parameter (λ) was identified using tenfold cross-validation, selecting the λ value that corresponded to the minimum mean cross-validated error within one standard error (1-SE criterion).Under this threshold, 12 variables with non-zero coefficients were retained, indicating their potential relevance for survival prediction (**Figures 2A, B**).Following variable selection, univariate Cox proportional hazards regression was performed on all 18 variables. Seven factors significantly associated with OS (p < 0.05) were identified: AJCC tumor stage, lymphovascular invasion, perineural invasion, and four systemic inflammatory indices—SII, NLR, PLR, and SIRI (**Table 2**).These variables were consistently identified by both LASSO regression and univariate Cox analysis and were therefore included as candidate predictors in the subsequent multivariate Cox regression model (**Figure 2C**).This dual-layered variable selection strategy ensured that the final model was constructed using robust and clinically meaningful parameters while minimizing the risk of overfitting. The integration of inflammation-based biomarkers with classical pathological factors offers a more comprehensive assessment framework for individualized risk stratification in bladder cancer patients.

**Table 2:** Univariable Cox regression analysis.

Variable		HR	95% confidence interval		*p*-value
Gender	Male	1.19	0.9	1.58	0.2274
	Ref:Female				
Age	>60	1.07	0.88	1.3	0.5108
	Ref:≤60				
Smoke	Yes	1.09	0.9	1.32	0.3668
	Ref:No				
Alcohol	Yes	1.07	0.88	1.3	0.4926
	Ref:No				
BMI	>25	1.09	0.87	1.36	0.4701
	Ref:≤25				
Diabetes	Yes	1.29	0.96	1.72	0.0894
	Ref:No				
Hypertensive	Yes	1.07	0.87	1.32	0.5172
	Ref:No				
Hematuria	Yes	0.86	0.67	1.12	0.2594
	Ref:No				
Renal_cyst	Yes	0.96	0.79	1.16	0.6553
	Ref:No				
Radical_prostatectomy	Yes	0.96	0.79	1.15	0.6377
	Ref:No				
Recurrence	Yes	1.28	0.81	2	0.2872
	Ref:No				
Nerve_invasion	Yes	3	2.48	3.62	**<0.001**
	Ref:No				
Vascular_tumor_thrombus	Yes	2.44	2.02	2.95	**<0.001**
	Ref:No				
AJCC_Stage	>II	3.52	2.91	4.26	**<0.001**
	Ref:≤II				
SII	>1005	3.12	2.53	3.84	**<0.001**
	Ref:≤1005				
NLR	>6.22	3.33	2.75	4.04	**<0.001**
	Ref:≤6.22				
PLR	>198	2.49	2.05	3.02	**<0.001**
	Ref:≤198				
SIRI	>2.53	2.81	2.32	3.41	**<0.001**
	Ref:≤2.53				

HR, hazard ratio; Bolded p-values indicate statistically significant correlations (p < 0.05).

### Survival analysis and multivariate Cox regression modeling

**Figure 3:**
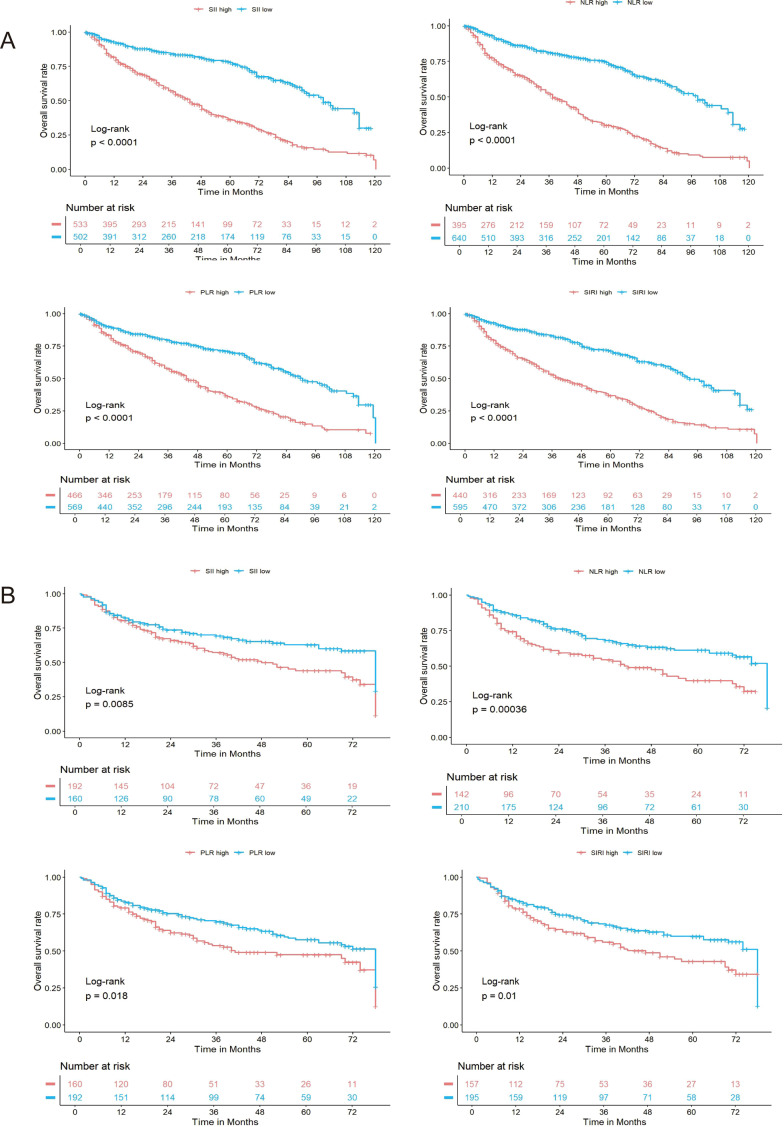
Kaplan–Meier survival curves based on systemic inflammatory indices in the training and validation cohorts. **(A)** Overall survival (OS) stratified by SII, NLR, PLR, and SIRI levels in the training cohort. Patients with elevated levels of each inflammatory index (red lines) had significantly worse OS compared to those with low levels (blue lines), as determined by the log-rank test (all p < 0.0001). **(B)** Kaplan–Meier curves for the same indices in the validation cohort demonstrated consistent results, with significantly lower OS in the high-level groups (all log-rank p-values < 0.05). The number of patients at risk over time is displayed below each plot.

Kaplan–Meier survival analysis and multivariate Cox proportional hazards modeling were conducted to further assess the prognostic significance of variables identified by LASSO and univariate Cox regression. Kaplan–Meier analysis demonstrated that higher systemic inflammatory indices (SII, NLR, PLR, and SIRI) were significantly associated with reduced overall survival in both the training and validation cohorts (log-rank test, all p < 0.05; **Figures 3A, B**).These findings underscore the association between a heightened systemic inflammatory response and unfavorable prognosis in bladder cancer.

**Figure 4:**
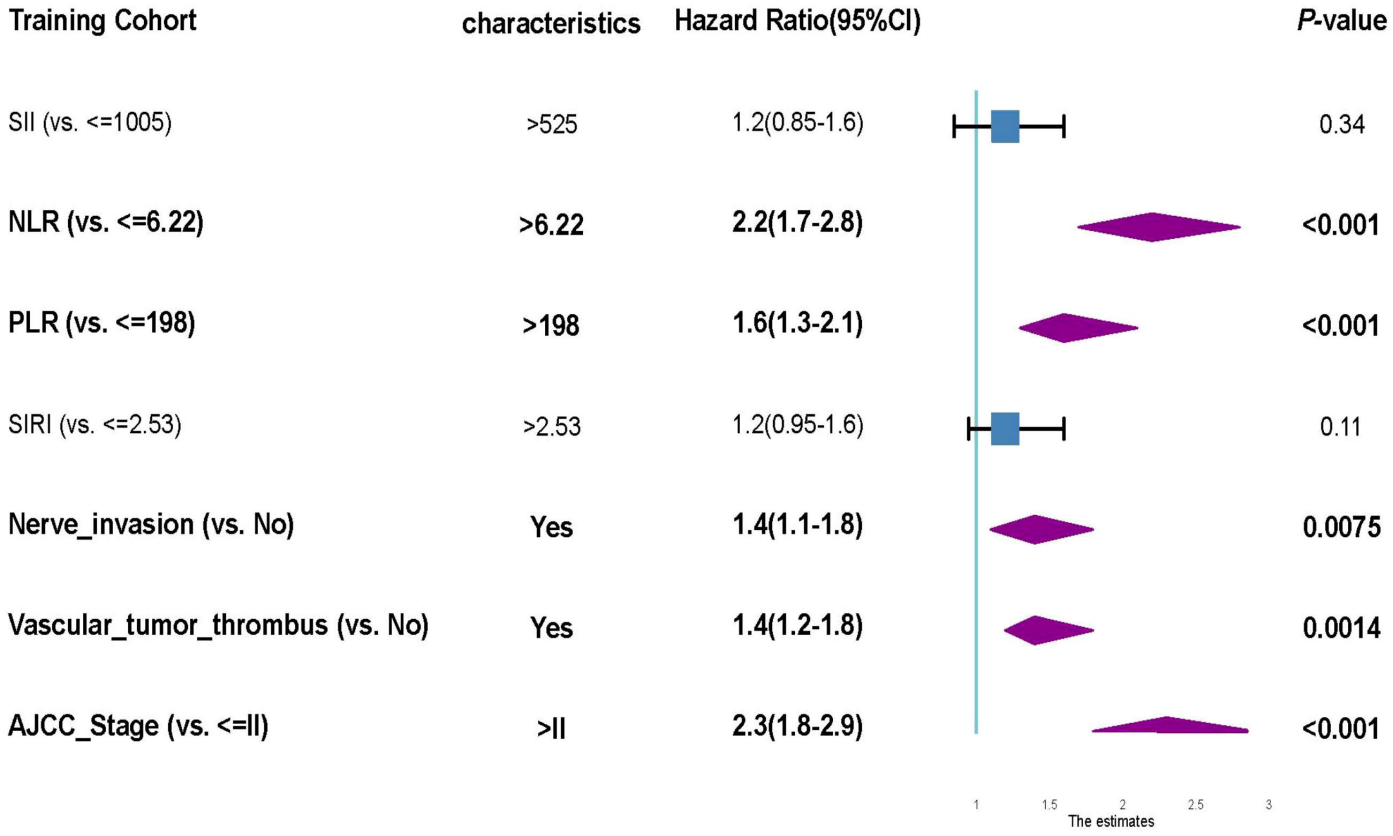
Forest plot of multivariate Cox proportional hazards regression for overall survival in the training cohort. The multivariate Cox model identified five independent predictors of poor overall survival: elevated NLR (>6.22), elevated PLR (>198), presence of perineural invasion, vascular tumor thrombus, and AJCC stage > II. Hazard ratios (HRs), 95% confidence intervals (CIs), and p-values are presented for each variable. SII and SIRI were not statistically significant in the multivariate model, suggesting limited independent prognostic value.

The multivariate Cox regression model incorporated all seven variables identified in the univariate analysis: AJCC tumor stage, lymphovascular invasion, perineural invasion, SII, NLR, PLR, and SIRI. The multivariate analysis revealed five independent adverse prognostic factors: NLR (HR = 2.2, 95% CI 1.7–2.8, p < 0.001), PLR (HR = 1.6, 95% CI 1.3–2.1, p < 0.001), perineural invasion (HR = 1.4, 95% CI 1.1–1.8, p = 0.007), lymphovascular invasion (HR = 1.4, 95% CI 1.2–1.8, p = 0.001), and AJCC stage > II (HR = 2.3, 95% CI 1.8–2.9, p < 0.001) (**Figure 4**). In contrast, SII and SIRI did not retain statistical significance after adjustment for covariates (p = 0.34 and p = 0.11, respectively), suggesting that their effects on survival may be confounded by other overlapping biological factors or collinearity with NLR and PLR. These findings underscore the significant and independent prognostic importance of NLR and PLR, easily obtained from standard blood tests, potentially indicating the equilibrium between tumor-promoting inflammation and host immune defense. These findings endorse the integration of specific systemic inflammation markers and key pathological variables to develop a clinically relevant prognostic model for bladder cancer patients undergoing radical cystectomy.

### Construction of the nomogram for individualized survival prediction

**Figure 5:**
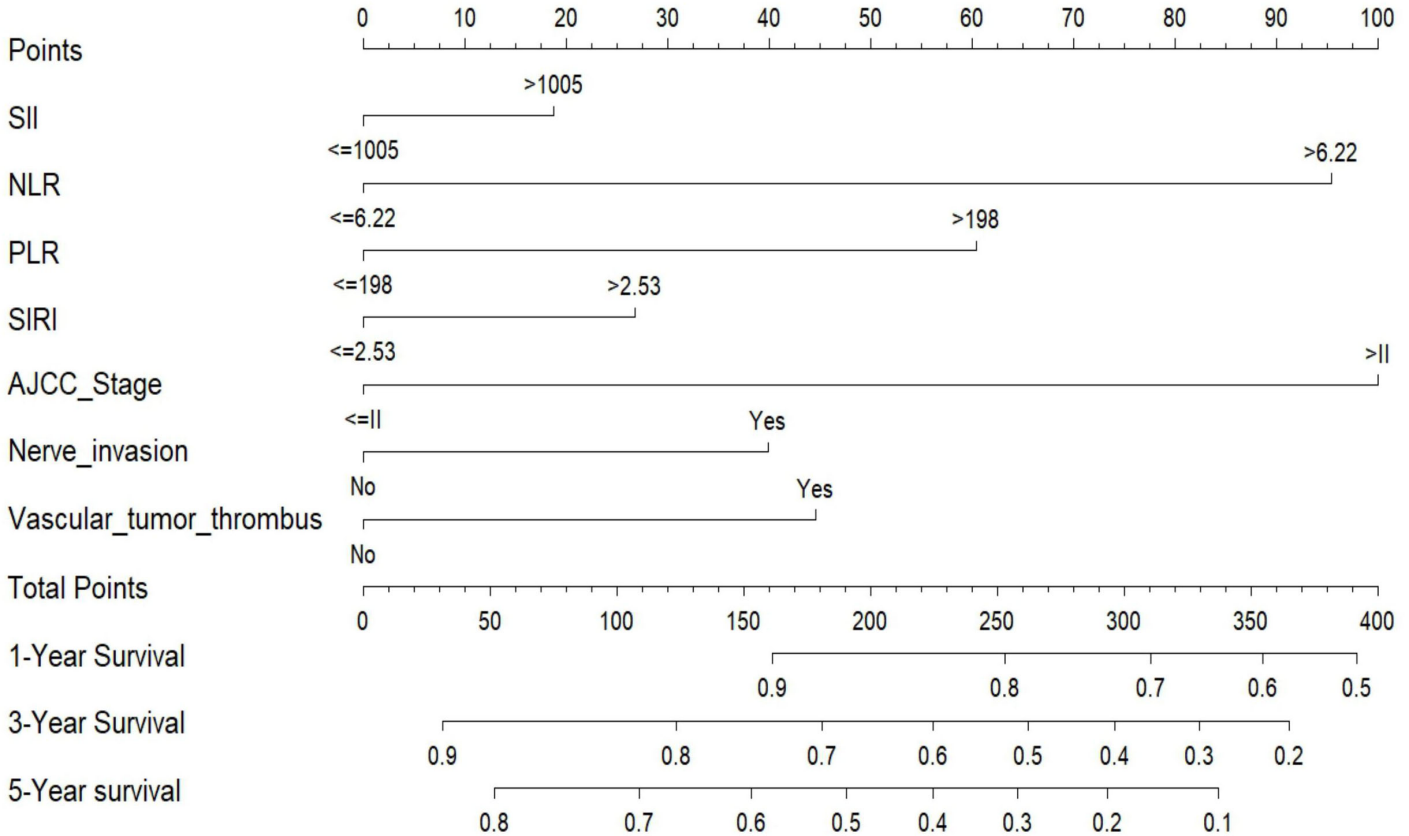
Nomogram for predicting 1-, 3-, and 5-year overall survival in patients with bladder cancer undergoing radical cystectomy. The nomogram was developed based on five independent prognostic factors identified by multivariate Cox regression analysis: neutrophil-to-lymphocyte ratio (NLR), platelet-to-lymphocyte ratio (PLR), perineural invasion, vascular tumor thrombus, and AJCC tumor stage. Although SII and SIRI were not statistically significant in the multivariate model, they were included in the nomogram due to their clinical relevance and univariate associations. For each patient, a total point score is calculated by summing the individual points for each variable. The corresponding 1-, 3-, and 5-year overall survival probabilities can be estimated using the scales at the bottom of the plot.

A prognostic nomogram was created using multivariate Cox regression analysis to predict 1-, 3-, and 5-year overall survival in bladder cancer patients undergoing radical cystectomy. The final model incorporated five independent prognostic factors: NLR, PLR, AJCC stage, perineural invasion, and lymphovascular invasion. Despite losing statistical significance in multivariate analysis, SII and SIRI showed prognostic value in univariate analysis and were included in the nomogram for their potential clinical relevance and contribution to the model’s overall performance (**Figure 5**). In the nomogram, each variable received a score proportional to its regression coefficient from the multivariate model. The total points for all variables can be summed to estimate the probabilities of 1-, 3-, and 5-year overall survival (OS) for individual patients. Among the included predictors, NLR, PLR, and AJCC stage contributed the most to the total score, reflecting their strong prognostic influence. Perineural invasion and lymphovascular invasion had moderate effects, while SII and SIRI contributed relatively lower weights but remained biologically and clinically relevant. This graphical tool provides a user-friendly and individualized method for preoperative survival prediction in clinical practice. By integrating both systemic inflammation markers and established pathological variables, the nomogram enables risk-adapted decision-making and personalized follow-up planning. High-risk patients identified by the model may benefit from intensified surveillance, early consideration of adjuvant therapy, or enrollment in clinical trials, while low-risk patients may avoid unnecessary interventions.

### Validation and performance evaluation of the nomogram model

**Figure 6:**
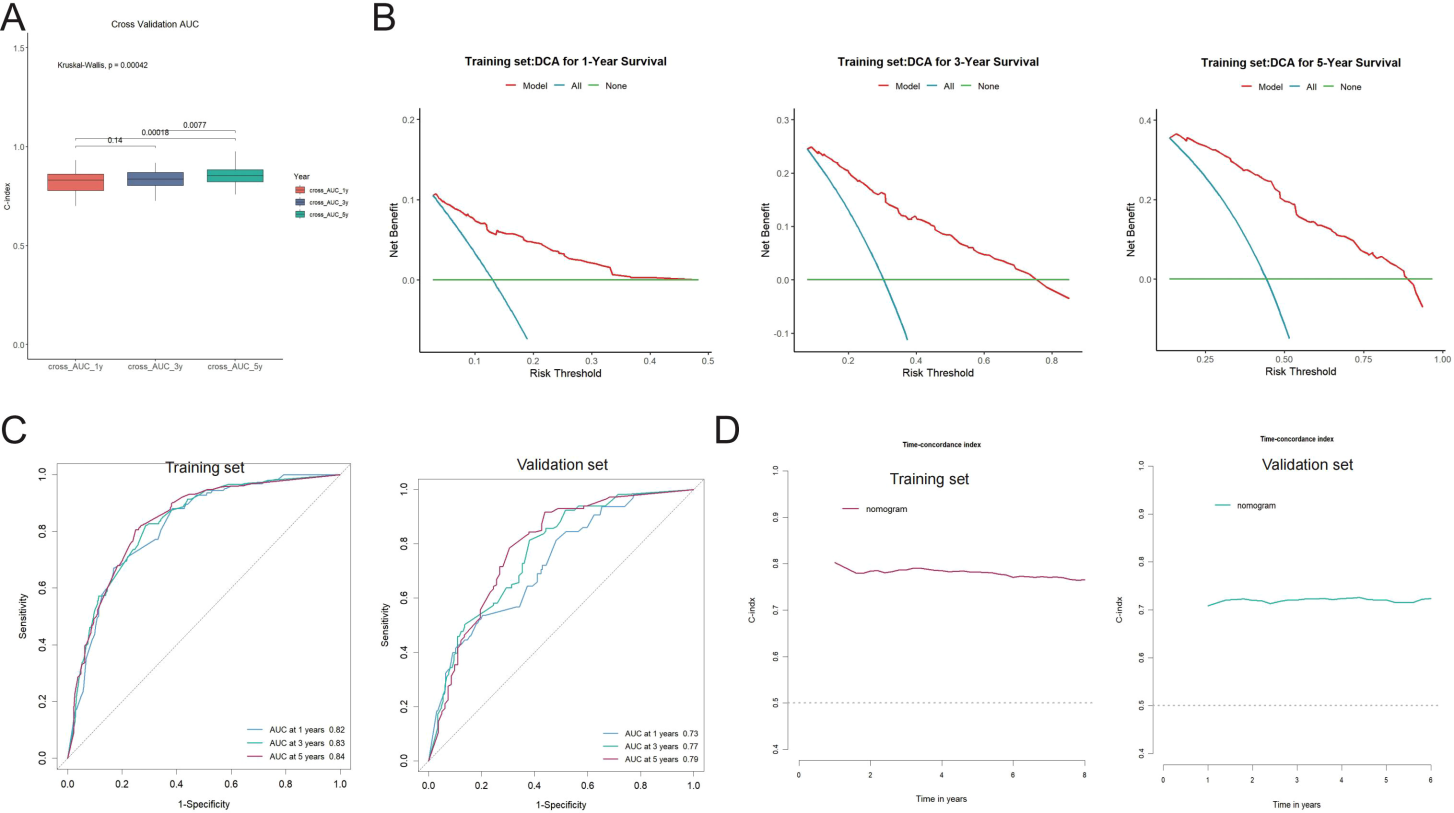
Validation of the nomogram model performance in the training and validation cohorts. **(A)** Tenfold cross-validation showing the distribution of AUC values for 1-, 3-, and 5-year overall survival predictions. The Kruskal-Wallis test indicates significant differences among time points (p < 0.05). **(B)** Decision curve analysis (DCA) for 1-, 3-, and 5-year survival in the training cohort. The red curve (model) shows greater net clinical benefit across a range of risk thresholds compared to the “all” and “none” strategies. **(C)** Time-dependent receiver operating characteristic (ROC) curves demonstrating the predictive performance of the nomogram. AUCs were 0.82, 0.83, and 0.84 at 1, 3, and 5 years in the training cohort, and 0.73, 0.77, and 0.79 in the validation cohort, respectively. **(D)** Harrell’s concordance index (C-index) over time in the training and validation cohorts, confirming stable model performance during follow-up.

**Figure 7:**
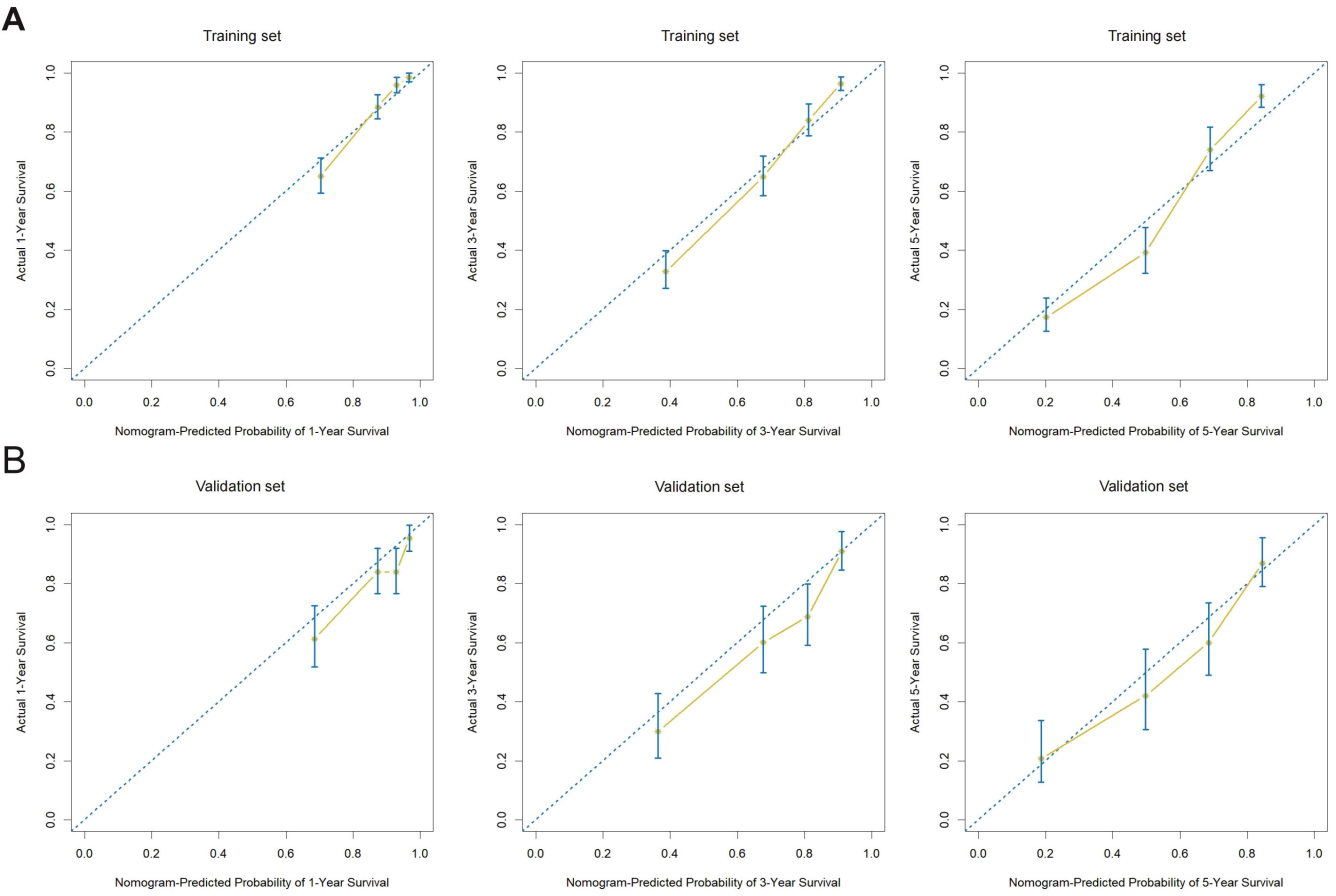
Calibration plots of the nomogram model for 1-, 3-, and 5-year overall survival **(A)** Calibration curves in the training cohort show excellent agreement between the predicted and actual survival probabilities at 1, 3, and 5 years, with points closely aligned to the ideal 45-degree line. **(B)** Calibration curves in the validation cohort demonstrate similar results, indicating good model calibration and generalizability across external data.

The prognostic nomogram’s predictive accuracy, calibration, and clinical utility were assessed in both the training and external validation cohorts. The model exhibited strong internal consistency in predicting 1-, 3-, and 5-year overall survival (OS) through tenfold cross-validation, with significant differences across time points (Kruskal–Wallis test, p < 0.05; **Figure 6A**). Decision curve analysis (DCA) validated the nomogram’s clinical utility, showing a higher net benefit over default strategies across various threshold probabilities, especially for 1- and 3-year survival predictions in the training cohort (**Figure 6B**).These results support the model’s utility in guiding individualized postoperative decision-making. Discriminatory performance was assessed using time-dependent receiver operating characteristic (ROC) curve analysis. In the training cohort, the AUC values for overall survival (OS) at 1, 3, and 5 years were 0.82, 0.83, and 0.84, respectively. The AUC values for the external validation cohort were 0.73, 0.77, and 0.79 (**Figure 6C**), demonstrating strong generalizability and discrimination. Model calibration was assessed using calibration plots that compared the predicted versus observed survival probabilities. In the training and validation cohorts, the calibration curves for 1-, 3-, and 5-year OS closely matched the 45-degree reference line, demonstrating strong agreement between predicted and actual outcomes (**Figures 7A, B**). Longitudinal evaluation of Harrell’s concordance index (C-index) showed consistent prognostic performance, averaging 0.78 in the training cohort and 0.72 in the validation cohort. These findings demonstrate that the nomogram consistently shows strong discrimination, calibration, and clinical utility across various independent datasets.

## Discussion

This multicenter retrospective study developed and validated a nomogram model to predict overall survival (OS) in bladder cancer patients undergoing radical cystectomy (RC). The model integrates preoperative inflammation-based scores (NLR, PLR, SII, SIRI) with key pathological features (AJCC stage, perineural invasion [PNI], and lymphovascular invasion [LVI]).The model demonstrated favorable discrimination and calibration across both internal and external cohorts, highlighting the prognostic relevance of systemic inflammatory responses as surrogate markers of tumor-host immune interplay.

Chronic inflammation is now widely recognized as a fundamental hallmark of cancer, involved in processes such as epithelial–mesenchymal transition (EMT), immune evasion, and angiogenesis (20, 21). Within the tumor microenvironment, inflammatory and immune cells secrete a spectrum of cytokines and chemokines that modulate immune suppression and tumor proliferation. Systemic inflammation alters peripheral blood cell profiles, and its intensity can be indirectly quantified using various inflammation-based indices (22–24). Numerous studies have confirmed that neutrophils, platelets, and lymphocytes each play pivotal roles in cancer biology (25). Neutrophils promote tumor progression via the secretion of VEGF, TGF-β, and proteolytic enzymes such as neutrophil elastase, MMP-9, and IL-8 (26–28). Platelets assist epithelial–mesenchymal transition and facilitate the extravasation of circulating tumor cells (CTCs) (29, 30). while lymphocytes orchestrate immune surveillance and cytotoxic responses (31).

Among these markers, NLR and PLR have emerged as simple yet reliable indicators of the balance between pro-tumor inflammation and anti-tumor immunity. Elevated NLR reflects increased neutrophilic inflammation and decreased lymphocyte-mediated immune defense, both of which contribute to a permissive environment for tumor spread (32, 33). Similarly, PLR elevation signals platelet activation, which is implicated in tumor cell adhesion, angiogenesis, and suppression of immune responses through cytokine release (34). In our analysis, both NLR and PLR remained independent predictors of OS in the multivariate Cox model, reinforcing their value in preoperative risk assessment. Notably, elevated levels of these indices have also been associated with poor response to BCG and immune checkpoint inhibitors in bladder cancer patients (5, 17).

SII and SIRI are recent composite indices that combine various immune components, potentially offering a more complete overview of systemic inflammation (16).Although previous reports suggest superior prognostic performance of SII/SIRI in some malignancies, our study did not find them to be independent predictors after adjusting for NLR and PLR. This discrepancy may be due to multicollinearity or overlapping biological pathways. Indeed, similar findings have been reported in other urologic cancers, where SIRI was more useful for treatment monitoring rather than preoperative prognostication (35).Nevertheless, we included SII and SIRI in the nomogram to enhance the model’s inclusiveness and potential for stratifying borderline-risk patients. From a clinical perspective, our model offers practical advantages. All parameters are routinely available from preoperative laboratory and pathological evaluations, making the model cost-effective and accessible even in resource-limited settings. Compared to models relying on genomic or radiomic data, this approach is more readily translatable to everyday clinical decision-making. Research indicates that clinicians favor inflammation-based models, which assist in treatment planning and monitoring (36, 37).Our nomogram, which combines inflammatory markers and pathological features, effectively predicts high-risk individuals who could benefit from intensive follow-up or adjuvant therapy, particularly in early identification. Despite the lack of statistical significance for SII and SIRI in the multivariate analysis of this study, this does not diminish their potential value in bladder cancer prognosis. Previous studies have indicated that these markers can provide useful information regarding systemic inflammatory responses, particularly in complex clinical contexts. We included them in the model to enhance its clinical inclusivity and to explore their potential application in bladder cancer patients. Although SII and SIRI did not serve as independent predictors in this study, their integration still contributes to improving the model’s multidimensional assessment capability. Future studies should continue to validate the clinical benefits of these markers, especially in different patient populations.

The inclusion of PNI and LVI further strengthened the model. These pathological features reflect microinvasive behavior and have been linked to adverse outcomes in numerous solid tumors. Meta-analyses have validated the prognostic significance of LVI across BCa subtypes (38), and PNI has been incorporated into scoring systems for colorectal and pancreatic cancers (39). In our cohort, the addition of PNI and LVI improved the C-index and highlighted their synergistic prognostic role alongside systemic inflammation. Combining inflammatory markers with microinvasive histological parameters may provide a more holistic representation of tumor aggressiveness and biological heterogeneity.

Emerging evidence highlights that systemic inflammation contributes to immune dysfunction within the tumor microenvironment (TME), particularly by promoting CD8^+^ T-cell exhaustion and expansion of immunosuppressive cells such as MDSCs. In bladder cancer, neutrophils—central to the NLR—can inhibit cytotoxic T cells via PD-L1–expressing NETs and ROS release, while platelets (reflected by PLR) secrete TGF-β and VEGF, promoting EMT and immune escape (28, 40). Elevated SII and SIRI may further indicate a chronically inflamed but immunologically suppressed TME. These mechanistic insights support our findings that high inflammation scores correlate with poor survival, suggesting potential as predictive markers for BCG efficacy or response to immune checkpoint inhibitors (41). Unlike prior models based on a single score, our nomogram uniquely integrates NLR, PLR, PNI, and LVI—capturing both systemic and microinvasive tumor features. The use of LASSO-based variable selection and robust external validation further enhances clinical applicability. Future studies should incorporate immune gene signatures, PD-L1 status, and spatial transcriptomics to refine the link between inflammation and immune phenotype. Integrating multi-omics data will enable deeper insight into immune-related heterogeneity and facilitate personalized immunotherapy strategies in bladder cancer.

This study, while robust, is not without limitations. First, as a retrospective design, potential biases cannot be entirely ruled out, even with strict inclusion criteria and multivariate adjustment. In particular, selection bias may arise due to the reliance on pre-existing data, which may not fully represent the entire bladder cancer patient population. Additionally, the data used in this study were sourced from two hospitals in Yunnan Province, China, which may limit the external validity of our findings. The geographical restriction may affect the generalizability of the results, as clinical characteristics and treatment responses may differ across different regions and countries. To enhance the model’s external validity and generalizability, future studies should expand the sample size and include more diverse patient populations from different regions or countries. To enhance the model’s external validity and generalizability, future studies should expand the sample size and include more diverse patient populations from different regions or countries, thus improving the model’s applicability in broader clinical settings. Prospective validation is warranted. Second, all inflammatory indices were measured at a single preoperative timepoint, limiting dynamic assessment of perioperative immune fluctuations. Third, molecular features such as luminal/basal subtype, FGFR3 mutation, tumor mutational burden (TMB), and immune gene signatures were not included in this model. These biomarkers may further refine the prediction of immunotherapy response and tumor heterogeneity. Emerging evidence supports the development of multimodal models that integrate molecular and immune-inflammation profiles for precision oncology (42, 43).The model proposed in this study combines inflammation-related markers (such as NLR, PLR, SII, SIRI) with traditional clinical-pathological features (such as AJCC stage, LVI, PNI), providing a more comprehensive prognostic tool for bladder cancer patients. Compared with existing bladder cancer prognostic nomograms, our model has significant advantages: first, the model integrates immune inflammation markers with traditional clinical-pathological features, thus assessing the prognosis of bladder cancer patients more comprehensively. Second, the model demonstrates higher predictive accuracy by jointly analyzing various clinical factors, showing stronger discriminatory ability across different patient groups. Through comparison with existing models, we emphasize that by integrating inflammation-related biomarkers, our model provides significant improvements in prognosis prediction, thereby highlighting its incremental contribution to enhancing the prognostic accuracy for bladder cancer patients (44).

## Conclusion

In this multicenter study, we established a clinically accessible nomogram integrating preoperative inflammatory scores and key pathological features to predict overall survival in patients with bladder cancer undergoing radical cystectomy. The model demonstrated reliable predictive performance and clinical utility across internal and external cohorts. Distinct from traditional staging tools, our nomogram captures both systemic immune-inflammatory status and tumor invasive behavior, offering a more nuanced assessment of patient prognosis. Its reliance on routine clinical data ensures broad applicability, even in resource-constrained settings. This tool can assist clinicians in identifying high-risk individuals who may benefit from intensified postoperative surveillance or adjunctive therapies, thereby enhancing precision in therapeutic decision-making. Future prospective studies and multi-omics integration are warranted to further refine its predictive power and explore its role in guiding immunotherapy and personalized care in bladder cancer.

## Data Availability

The datasets presented in this study can be found in online repositories. The names of the repository/repositories and accession number(s) can be found in the article/supplementary material.
