# Correction: Preoperative inflammation-based immune prognostic nomogram in bladder cancer: a multicenter study

**DOI:** 10.3389/fonc.2026.1787982

**Published:** 2026-02-20

**Authors:** Runlin Feng, Jian Zhang, Yanping Tao, Jian Hou, Wenlin Tai

**Affiliations:** 1 Department of Pathology, The Second Affiliated Hospital, Kunming Medical University, Kunming, Yunnan, China; 2 Yunnan Key Laboratory of Stomatology & Department of Oral and Maxillofacial Surgery, The Affiliated Stomatology Hospital, Kunming Medical University, Kunming, Yunnan, China; 3 Department of Emergency Medicine, Kunming Third People’s Hospital, Kunming, Yunnan, China; 4 Department of Urology, The First Affiliated Hospital, Kunming Medical University, Kunming, Yunnan, China; 5 Department of Laboratory Medicine, The Second Affiliated Hospital, Kunming Medical University, Kunming, Yunnan, China

**Keywords:** bladder cancer, radical cystectomy, systemic inflammation, NLR, prognostic nomogram, overall survival, immune microenvironment, tumor inflammation

There was a mistake in the order of the figures in the published article. Specifically:

**Figure 2** should be placed where **Figure 3** currently is.

**Figure 2:**
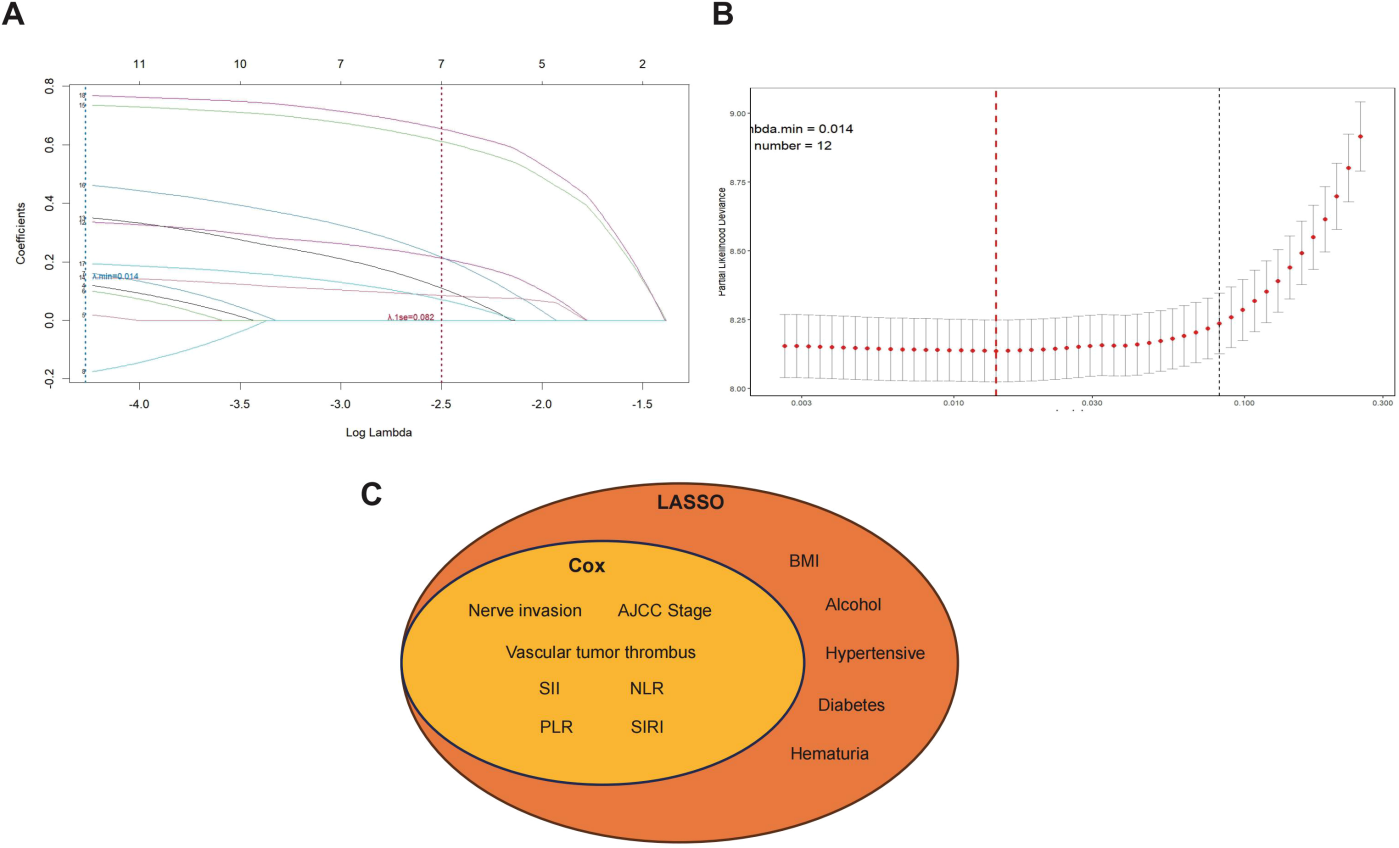
Selection of prognostic features using LASSO regression and univariate Cox analysis. **(A)** LASSO coefficient profiles of 18 candidate variables plotted against the log(λo sequence. Each curve represents the trajectory of a coefficient as a function of the regularization parameter. **(B)** 10-fold cross-validation for tuning parameter selection in the LASSO model. The optimal λ value was determined using the 1-standard-error rule, yielding 12 variables with non-zero coefficients. **(C)** Venn diagram illustrating the overlap between prognostic features identified via LASSO regression and univariate Cox analysis. Seven variables, including nerve invasion, vascular tumor thrombus, AJCC stage, SII, NLR, PLR, and SIRI, were retained in both models and selected as key predictors for multivariate analysis.

**Figure 3** should be placed where **Figure 4** currently is.

**Figure 3:**
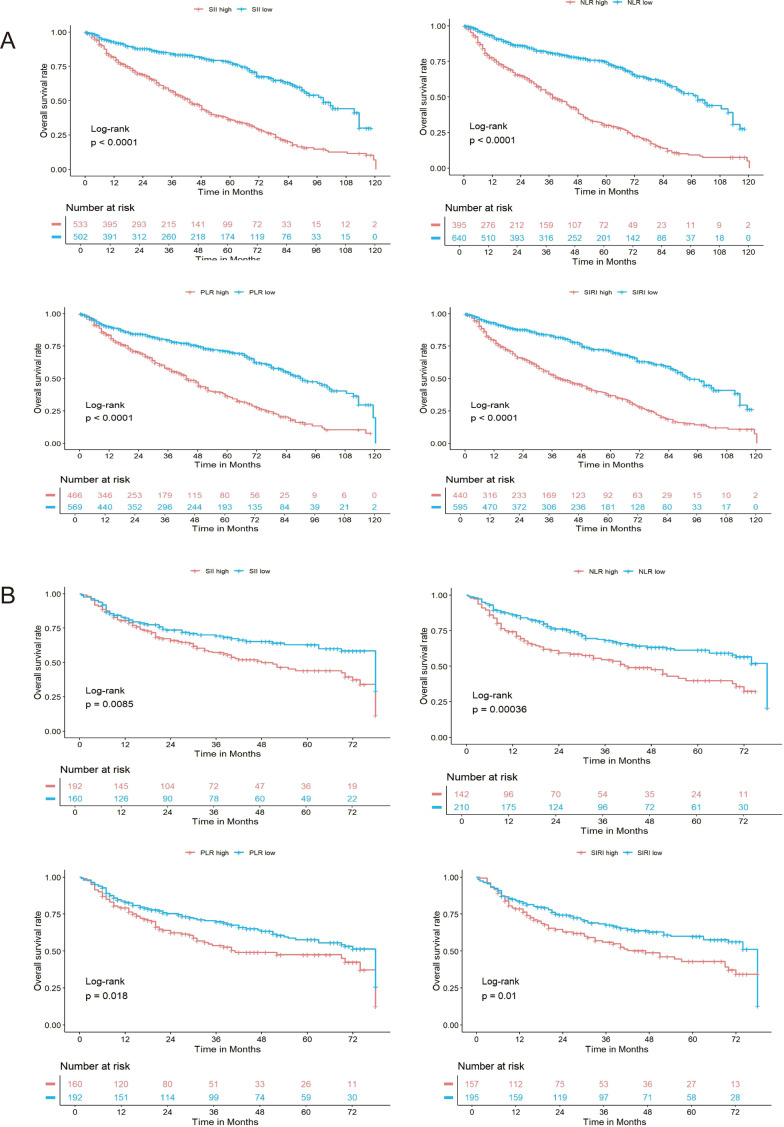
Kaplan–Meier survival curves based on systemic inflammatory indices in the training and validation cohorts. **(A)** Overall survival (OS) stratified by SII, NLR, PLR, and SIRI levels in the training cohort. Patients with elevated levels of each inflammatory index (red lines) had significantly worse OS compared to those with low levels (blue lines), as determined by the log-rank test (all p < 0.0001). **(B)** Kaplan–Meier curves for the same indices in the validation cohort demonstrated consistent results, with significantly lower OS in the high-level groups (all log-rank p-values < 0.05). The number of patients at risk over time is displayed below each plot.

**Figure 4** should be placed where **Figure 5** currently is.

**Figure 4:**
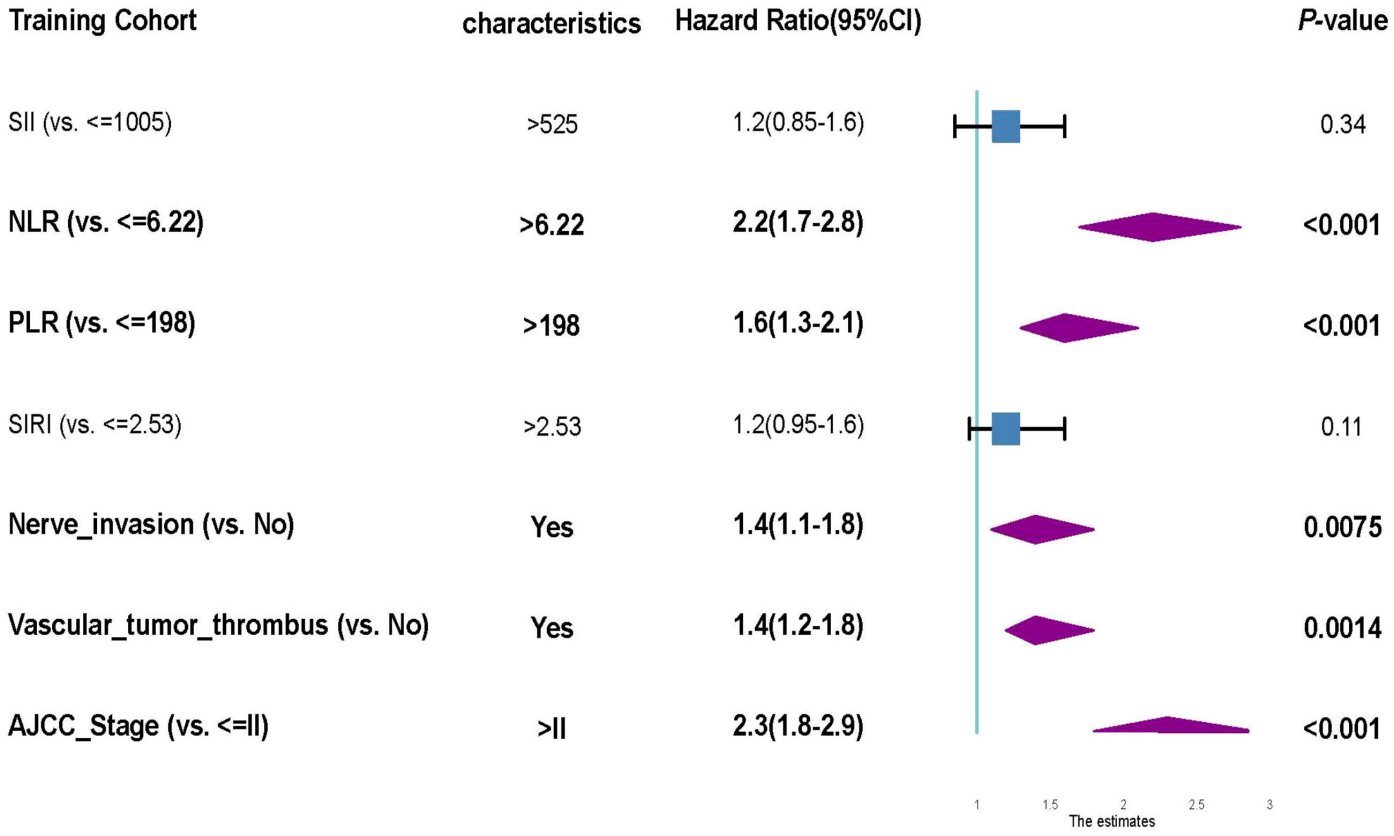
Forest plot of multivariate Cox proportional hazards regression for overall survival in the training cohort. The multivariate Cox model identified five independent predictors of poor overall survival: elevated NLR (>6.22), elevated PLR (>198), presence of perineural invasion, vascular tumor thrombus, and AJCC stage > II. Hazard ratios (HRs), 95% confidence intervals (CIs), and p-values are presented for each variable. SII and SIRI were not statistically significant in the multivariate model, suggesting limited independent prognostic value.

**Figure 5** should be placed where **Figure 7** currently is.

**Figure 5:**
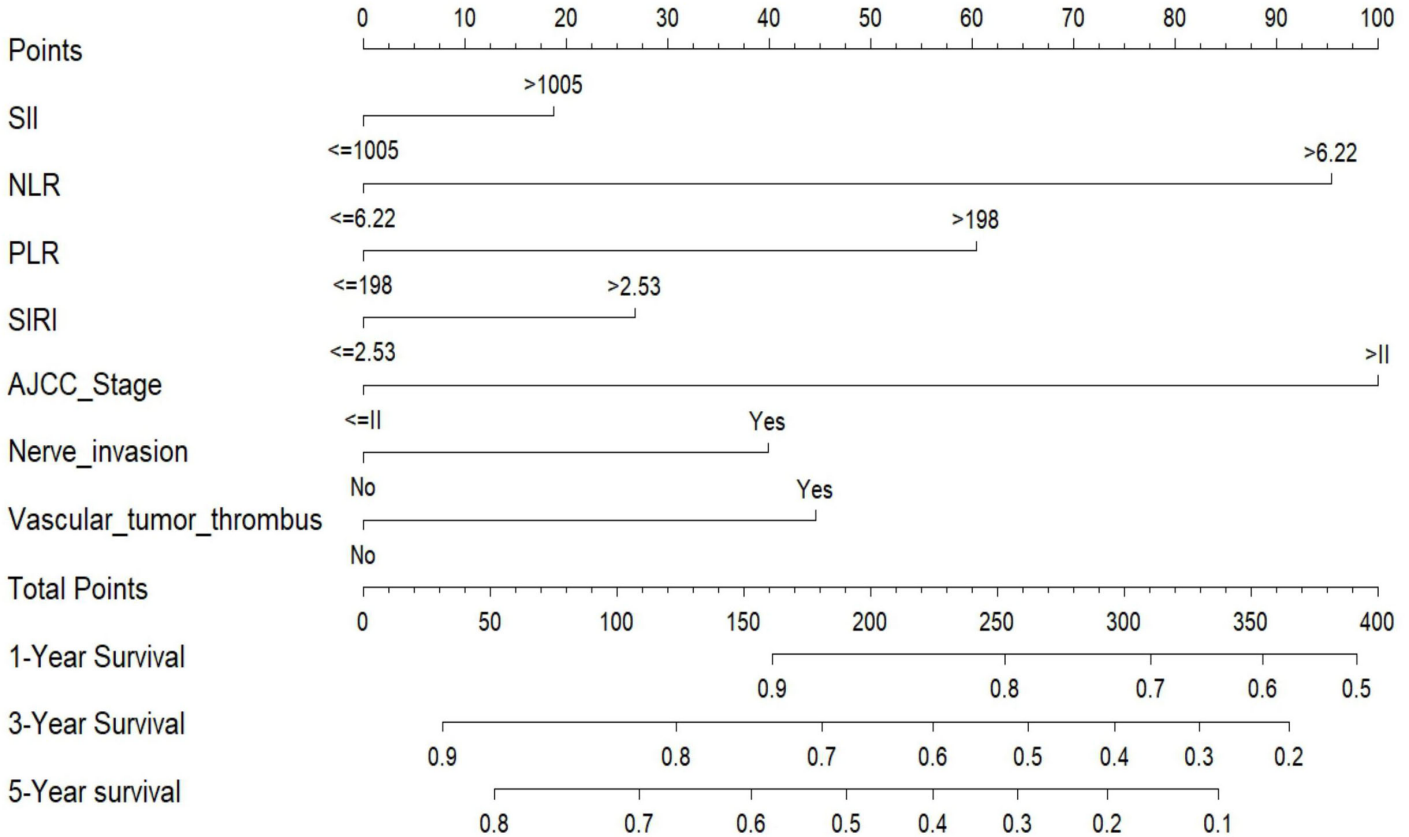
Nomogram for predicting 1-, 3-, and 5-year overall survival in patients with bladder cancer undergoing radical cystectomy. The nomogram was developed based on five independent prognostic factors identified by multivariate Cox regression analysis: neutrophil-to-lymphocyte ratio (NLR), platelet-to-lymphocyte ratio (PLR), perineural invasion, vascular tumor thrombus, and AJCC tumor stage. Although SII and SIRI were not statistically significant in the multivariate model, they were included in the nomogram due to their clinical relevance and univariate associations. For each patient, a total point score is calculated by summing the individual points for each variable. The corresponding 1-, 3-, and 5-year overall survival probabilities can be estimated using the scales at the bottom of the plot.

**Figure 7** should be placed where **Figure 2** currently is.

**Figure 6:**
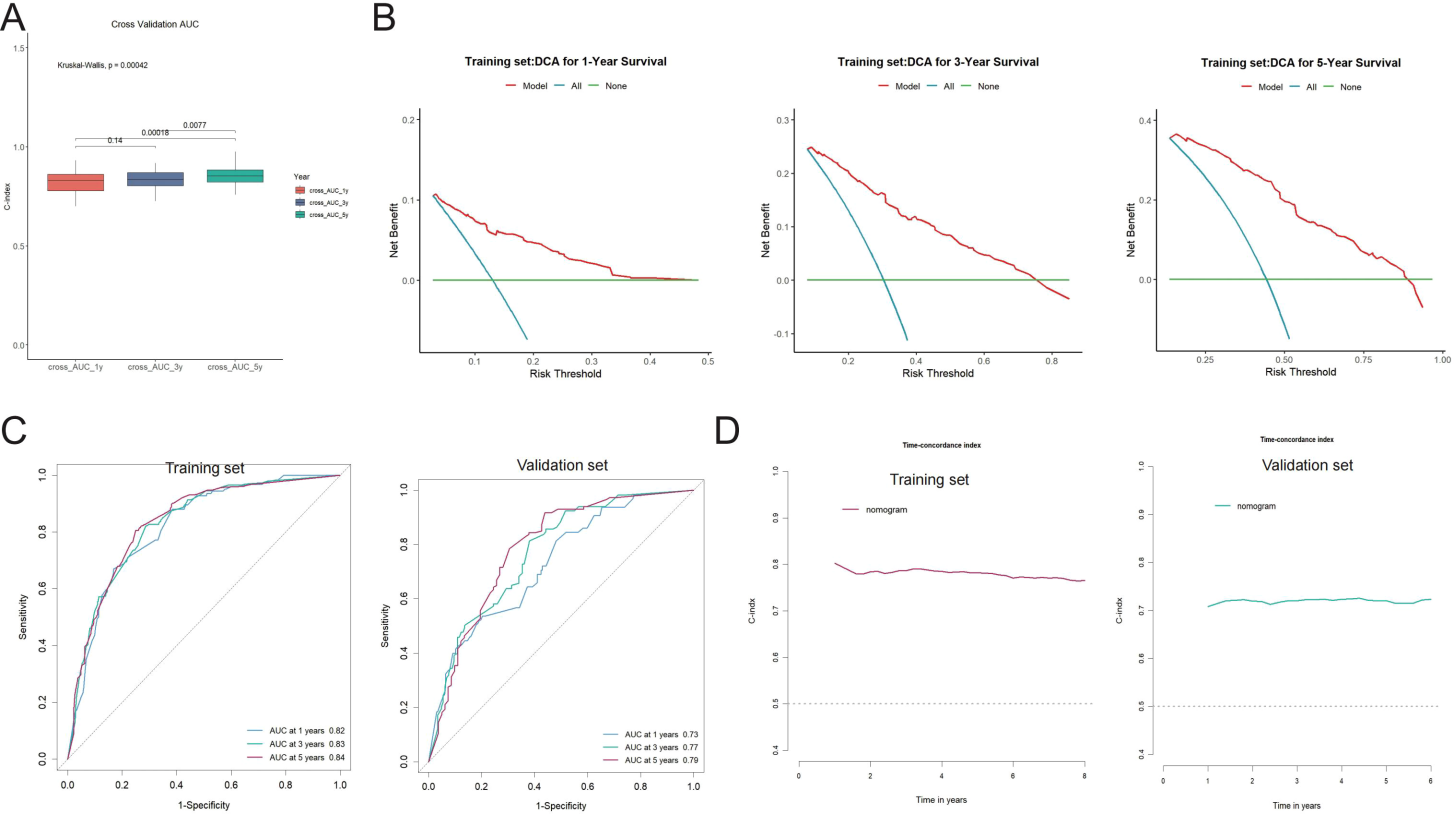
Validation of the nomogram model performance in the training and validation cohorts. **(A)** Tenfold cross-validation showing the distribution of AUC values for 1-, 3-, and 5-year overall survival predictions. The Kruskal-Wallis test indicates significant differences among time points (p < 0.05). **(B)** Decision curve analysis (DCA) for 1-, 3-, and 5-year survival in the training cohort. The red curve (model) shows greater net clinical benefit across a range of risk thresholds compared to the “all” and “none” strategies. **(C)** Time-dependent receiver operating characteristic (ROC) curves demonstrating the predictive performance of the nomogram. AUCs were 0.82, 0.83, and 0.84 at 1, 3, and 5 years in the training cohort, and 0.73, 0.77, and 0.79 in the validation cohort, respectively. **(D)** Harrell’s concordance index (C-index) over time in the training and validation cohorts, confirming stable model performance during follow-up.

**Figure 7:**
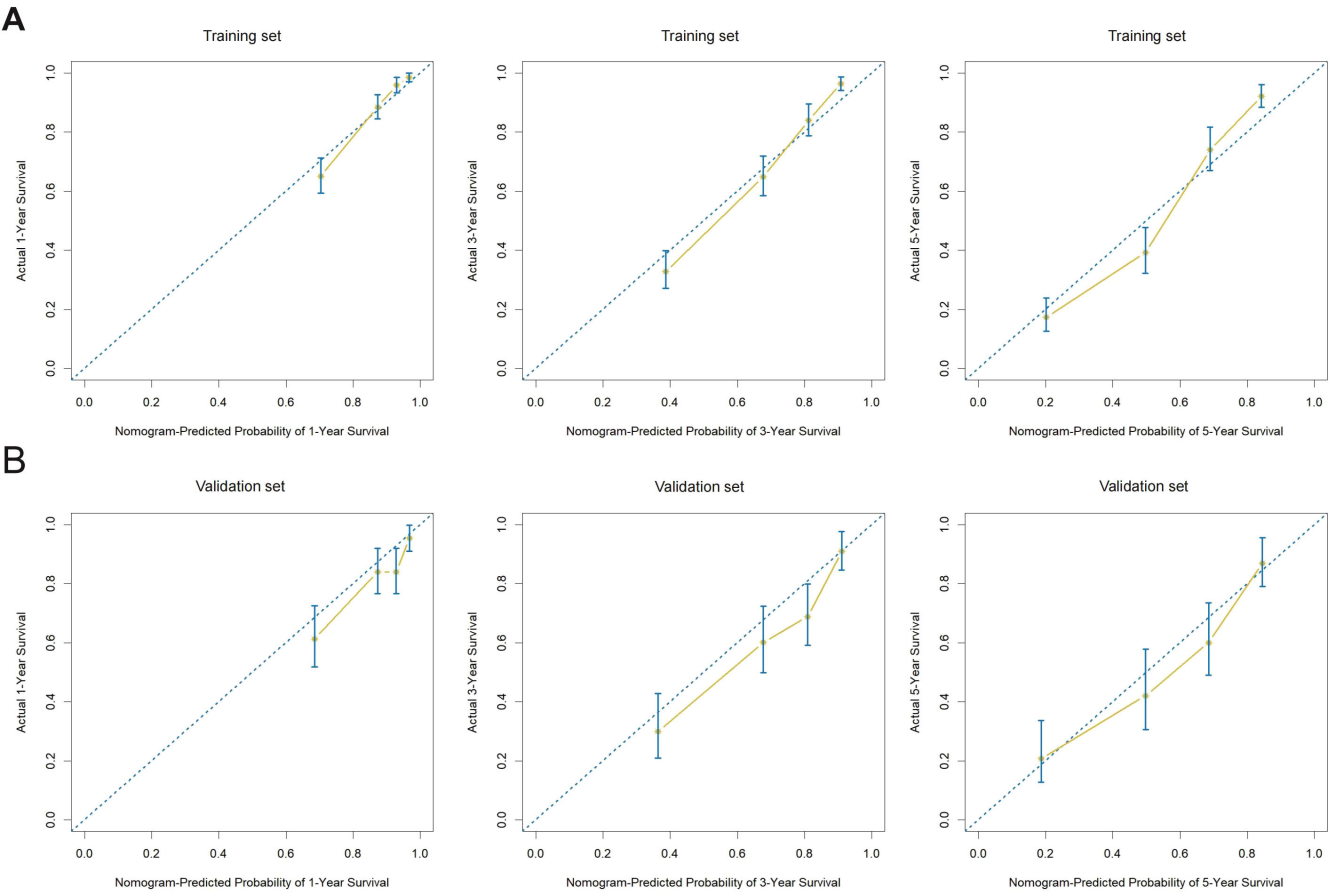
Calibration plots of the nomogram model for 1-, 3-, and 5-year overall survival **(A)** Calibration curves in the training cohort show excellent agreement between the predicted and actual survival probabilities at 1, 3, and 5 years, with points closely aligned to the ideal 45-degree line. **(B)** Calibration curves in the validation cohort demonstrate similar results, indicating good model calibration and generalizability across external data.

**Figure 6** is correct and does not need to be changed.

The images have now been reordered correctly.

The images were in the wrong order in the published version of the paper, while the figure captions are correct and have not been changed. The order has now been corrected as described above.

The original version of this article has been updated.

